# Enhancing removal efficiency of anionic dye (Cibacron blue) using waste potato peels powder

**DOI:** 10.1038/s41598-020-79069-5

**Published:** 2021-01-22

**Authors:** Kahina Bouhadjra, Wahiba Lemlikchi, Azedine Ferhati, Samuel Mignard

**Affiliations:** 1 Laboratory of Applied Chemistry and Chemical Engineering (LCAGC), University of Tizi-Ouzou, Tizi-Ouzou, Algeria; 2 High National School of Public Works (ENSTP), El Kouba, Algiers, Algeria; 3 University of Algiers 1, Algiers, Algeria; 4 Laboratory Chemistry and Environmental Chemistry (LCCE), University of Batna 1, Batna, Algeria; 5 Institute of Chemistry of Environments and Materials of Poitiers (IC2MP), Poitiers, France

**Keywords:** Environmental sciences, Chemistry, Engineering, Materials science, Nanoscience and technology

## Abstract

In the present study, the potato peel waste (PP) was used for the removal of the anionic dye Cibacron Blue P3R from an aqueous solution, activated with phosphoric acid (PPa) and calcined at 800 °C (PPc). The materials were characterized by Scanning Electron Microscope, Energy dispersive X-ray analysis and Fourier Transform Infrared Spectroscopy. The effects of various experimental parameters (pH, dye concentration, contact time) were also studied. The experimental results have shown that PPc has a greater capacity compared to pp and ppa. The capacity of PP bio-char (PPc) is 270.3 mg g^−1^ compared to PP (100 mg g^−1^) and PPa (125 mg g^−1^). Equilibrium experiments at 180 min for all materials were carried out at optimum pH (2.2): 76.41, 88.6 and 94% for PP, PPa and PPc respectively; and the Langmuir models agreed very well with experimental data. The ability of sorbent for the sorption of CB dye follows this order: calcined > activated > native materials. Potato peel biochar (PPc) can be considered a promising adsorbent for removing persistent dyes from water.

## Introduction

The increasing use of dyes in modern industries such as food, plastics, rubber, textile and cosmetics has raised environmental concerns about their stability to light and oxidizing agents. This has encouraged intensive research for inexpensive and easily available adsorbents to remove these pollutants from water.

One of these major problems is the generation of large amounts of colored wastewaters from textile dyeing. These dyes are synthetics and can be classified into diverse groups: acidic, reactive, direct, basic and azoic. Anionic dyes include reactive dyes, direct dyes and acid dyes, which can cause severe organic and color pollution in the water environment. Therefore, their removal by sorption or other process is a necessity for the protection of the environment.

There are conventional methods for removing dyes from wastewater such as coprecipitation^1–7^ and electrochemical techniques^8, 9^.

Biosorption, can be defined as the ability of biological materials to accumulate textile dyes from wastewater through metabolically mediated or physicochemical pathways of uptake ^10–14^.

This work presents an investigation of the use of agriculture residues of potato peels (PP) for adsorption of an anionic dye CB (Reactive blue 49 or Cibacron blue P3R), species present in the wastewaters from textile industries. This biomass is activated using phosphoric acid to enhance its adsorption efficiency. The bio-char is prepared at 800 °C under inert media. Optimal operating conditions were examined in this work. Indeed, the extent of adsorption was considered as a function of the pH of the solution, the contact time and the initial dye concentration.

## Materials and methods

### Adsorbate

Reactive blue 49 or Cibacron blue P3R (CB) was the anionic dye used in this study. It was supplied by DBK textile industry (Algeria) and used without any purification. The structure of Blue Cibacron (BC), and the chemical characteristics are consolidated in Table 1.

**Table 1:** Chemical structure and general characteristics of Cibacron blue CB.


Chemical name	Blue reagent 49
Chemical formula	C_32_H_23_ClN_7_Na_3_O_11_S_3_
Molecular weight (g/mole)	878 g/mol
Solubility	Soluble in water
Dyeing class	Reactive dye
Trading name	Cibacron blue P-R3
% Dye purity	92%

Colored solutions were prepared by dissolving required quantity of CB in distilled water to produce a stock solution of 1000 mg L^−1^ with a pH ~ 6.5. Adsorption studies for the evaluation of PP adsorbent for the removal of CB dye from aqueous solutions were carried out in a series of 100 mL flasks using a batch contact adsorption method. The concentrations of dye was determined from its characteristic UV–Vis absorbance with the calibration method. UV–Vis spectrophotometer Shimadzu/Model UV-1601PC was used for measurement of absorbance of CB solutions at λ_max_ = 625 nm.

### Adsorbents

Potato peels (Solanum tuberosum), supplied as wastes from restaurants, were cut into smaller pieces, thoroughly washed with tap water and left in open air for several days and then dried at 70 °C. The peels were crushed and milled into different particle sizes in the range of 0.08–0.63 mm. the potato peels powder (PP) was washed several times with distilled water until the wash water pH became identical to that of the distilled water (nearly 7). Resulting PP, was kept in glass bottles for use in the adsorption study. Chemical activation of the dry biomass was carried out using H_3_PO_4_ acid (analytical grade putity ≥ 98%). 25 g of dry potato powder precursor impregnated with 250 mL of 1 M solutions of H_3_PO_4_ at room temperature and kept under stirring for 18 h. The powder was washed abundantly with distilled water to a constant pH and then dried at 105 °C, to constant weight (for about 24 h). During the modification process, the dried activated biomass (PPa) was modified structurally by large quantities of new functional groups and new adsorption sites, which could increase the adsorption capacity. We employed dry potato powder to produce (PPc) with steam as a physical activation agent. The experiments were performed in two stages; i.e. the pyrolysis was performed in a quartz reactor at 800 °C for 60 min under nitrogen flow (15 mL min^−1^) and a heating rate of 27 °C / min, and then the physical activation of char was performed (800 °C) for 30 min under steam flow (10 g min^−1^). Pyrolysis was conducted in a tube furnace (PROTHERM PTF 12/75/600 Model).

Elemental analyses of potato peels powder (PP) was performed using organic elemental analyzer (CHONS-FLASH 2000 Themo Scientific). The surface functional groups were determined automatically using a titration method. Potentiometric measurements were taken with an automatic titrator (Titrino plus 848, Metrhm), 0.1 g of the PP was placed in 75 mL of NaNO_3_ solution (0.01 mol L^−1^) as a supporting electrolyte and stirred overnight. The solution was titrated with NaOH (0.1 mol L^−1^) under N_2_ saturation. The titration was carried out over a wide range of pH. The total Q_surf_ (mmol g^−1^), was determined as a function of pH by a numerical program SAIEUS (Solution of Adsorption Integral Equation Using Spline) after fitting. The morphology of the PP adsorbent was investigated using SEM images obtained from a (HITACHI SC-2500 model). The elemental compositions was analyzed by EDX (energy dispersive X-ray analysis). The thermogravimetric analyses (DTG) of the PP adsorbent were conducted using about 10 mg of the materials, under a nitrogen atmosphere from 25 to 700 °C in an SDT 2960 thermoanalyzer. FTIR spectra were recorded in the spectral range 4000–500 cm^−1^ on a ThermoNickel 6700 FTIR spectrometer. Buffered solutions at pH ~ 2.2 were used since the adsorption of anionic dyes is maximum at this pH value^15^.

The adsorption kinetic experiments were performed by batch procedures in a thermostat shaker (120 oscillations per min.) at 25 ± 0*.*1 °C, using 20 mg L^−1^ in aqueous dye solutions. This concentration was chosen to verify the performance of the materials in removing CB dye from diluted aqueous solutions. After 4 h of agitation, equilibrium condition was attained and samples were taken and filtered to determine concentration of the dye left in the solution with the aid of UV–Vis spectrophotometer at maximum wavelength λ_max_ = 625 nm. The adsorption capacity q (mg g^−1^) and percentage removal were obtained using (Eq. 1) and (Eq. 2) respectively:

1 $${\text{q}}_{{\text{e}}} = \frac{{\left( {{\text{C}}_{0} - C_{t} } \right) \times {\text{V}}}}{{\text{m}}}$$

2 $${\text{\% Removel}} = \frac{{{\text{C}}_{0} - C_{t} }}{{{\text{C}}_{0} }} \times 100{ }$$

where ${\text{C}}_{0}$ and $C_{t}$ (mg l^−1^) are the adsorbate concentrations at the initial time and at a given time t, respectively. ${\text{V}}$ is the experimental volume expressed in liters and m is the adsorbent mass expressed in grams.

### Effect of initial pH

The effect of the initial pH on removal of CB dye was investigated at different pH values (2, 4, 6, 8 and 10). The initial concentration of CB was fixed to 30 mg L^−1^ and adsorbent dose of 2 g L^−1^ for (PP and PPa), and of 0.6 g L^−1^ for (PPc). The samples were shaken for 2 h in a thermostat shaker at 25 ± 0.1 °C.

### Kinetic study

Kinetic experiments were performed by mixing 2 g L^−1^ of (PP and PPa) and 0.6 g L^−1^ of PPc, with 50 mL of dye solution (${\text{C}}_{0}$ = 30 mg L^−1^). The suspensions were shaken for 180 mn at pH = 2.2 (optimum pH found as will be discussed later) in a thermostat shaker at 25 ± 0.1 °C. Samples were collected at fixed intervals (5 min–180 min). The experimental kinetic data were fitted to two kinetic models; pseudo-first^16^ (Eq. 3) and second order^17^ (Eq. 4).

3 $$\ln \left( {{\text{q}}_{{\text{e}}} - q_{t} } \right) = \ln \left( {{\text{q}}_{{\text{e}}} } \right) - \frac{{{\text{K}}_{1} }}{2,303}t$$

4 $$\frac{t}{{q_{t} }} = \frac{1}{{{\text{k}}_{{2{ }}} {\text{q}}_{{\text{e}}}^{2} }} + \frac{1}{{{\text{q}}_{{\text{e}}} }}t$$

where $q_{t}$ is the amount of dye sorbed (mg g^−1^) at a given time, k_1_ (min^−1^) and k_2_ (g mg^−1^ mn^−1^) are the first and second-order-rate constants of sorption, respectively.

### Effect of initial dye concentration—isotherms

The effect of the initial dye concentration on equilibrium was observed by mixing 2 g L^−1^ of PP and PPa, 0.6 g L^−1^ of PPc with 50 mL of dye solutions of varying initial concentrations (C_0_ = 10–300 mg L^−1^). The suspensions were shaken for 4 h at optimum pH in a thermostat shaker at 25 ± 0*.*1 °C. The experimental equilibrium data were fitted to the Langmuir (Eqs. 5, 6)^18^, and Freundlich (Eq. 7)^19^ :

5 $${\text{Langmuir I}} \; \frac{1}{{q_{e} }} = \frac{1}{{{\text{q}}_{{{\text{max}}}} }} + \frac{1}{{{\text{q}}_{{{\text{max}}}} \cdot {\text{ K}}_{{\text{L }}} \cdot C_{e} }}$$

6 $${\text{Langmuir II}} \; \frac{{C_{e} }}{{q_{e} }} = \frac{1}{{{\text{q}}_{{{\text{max}}}} {\text{K}}_{{\text{L }}} }} + \frac{{C_{e} }}{{{\text{q}}_{{{\text{max}}}} }}$$

7 $$Ln_{{q_{e} }} = \frac{1}{{\text{n}}}LnC_{e} + {\text{ LnK}}_{{\text{f}}}$$

where ${\text{q}}_{{{\text{max}}}}$ (mg g^−1^) is the maximum amount of adsorption; ${\text{ K}}_{{\text{L }}}$ (L mg^−1^) is the Langmuir adsorption equilibrium constant. ${\text{K}}_{{\text{f}}}$ and 1/n were the constants; ${\text{K}}_{{\text{f}}}$ is a constant relating to the adsorption capacity. While 1/n is related to the intensity of adsorption or surface heterogeneity, becoming more heterogeneous as its value gets closer to zero. A value of 1/n < 1 is indicative of a Langmuir isotherm, while 1/n > 1 indicates a cooperative adsorption^19^. q_e_ is dye concentration at equilibrium onto biosorbent (mg g^−1^). $C_{e}$ is dye concentration at equilibrium in solution (mg l^−1^). The shape of the Langmuir isotherm can also be expressed in terms of separation factor (${\text{R}}_{{\text{L}}}$), which is given as follows^20^:

8 $${\text{R}}_{{\text{L}}} = \frac{1}{{1 + {\text{K}}_{{\text{L}}} .{\text{C}}_{0} }}$$

where ${\text{ K}}_{{\text{L }}}$ is Langmuir constant (L mg^−1^) related to the affinity of binding sites and the free energy of sorption and C_0_ is the initial concentration in the solution (mg L^−1^). For favorable adsorption, 0 < ${\text{R}}_{{\text{L}}}$ < 1, while ${\text{R}}_{{\text{L}}}$ > 1, ${\text{R}}_{{\text{L}}}$ = 1 and ${\text{R}}_{{\text{L}}}$ = 0 describe unfavorable, linear and irreversible adsorption, respectively.

## Results and discussion

### Surface proprieties and EDX analysis

**Figure 1:**
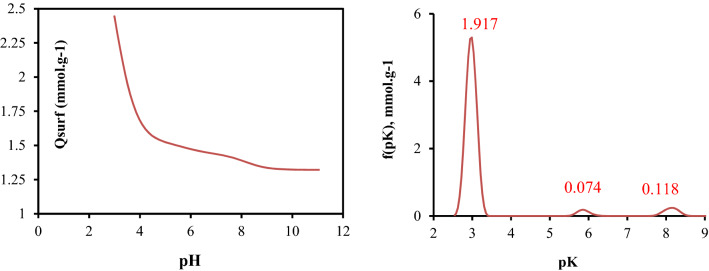
Potentiometric titration curve for PP.

Although the structure of biomaterials is mainly made from cellulose, hemicelluloses and lignin and are generally similar in structure, the type of functional groups and their acid and basic proprieties are different^17, 29^. The acidic and basic surface groups were estimated after potentiometric titration measurements and the results are presented in Fig. 1.

It is obvious that the PP is acid, as the surface pH measurements confirmed the presence of more oxygenated surface groups. This result further confirmed the nature of the PP surfaces, which enhanced the adsorption of CB from the solution.

**Figure 2:**
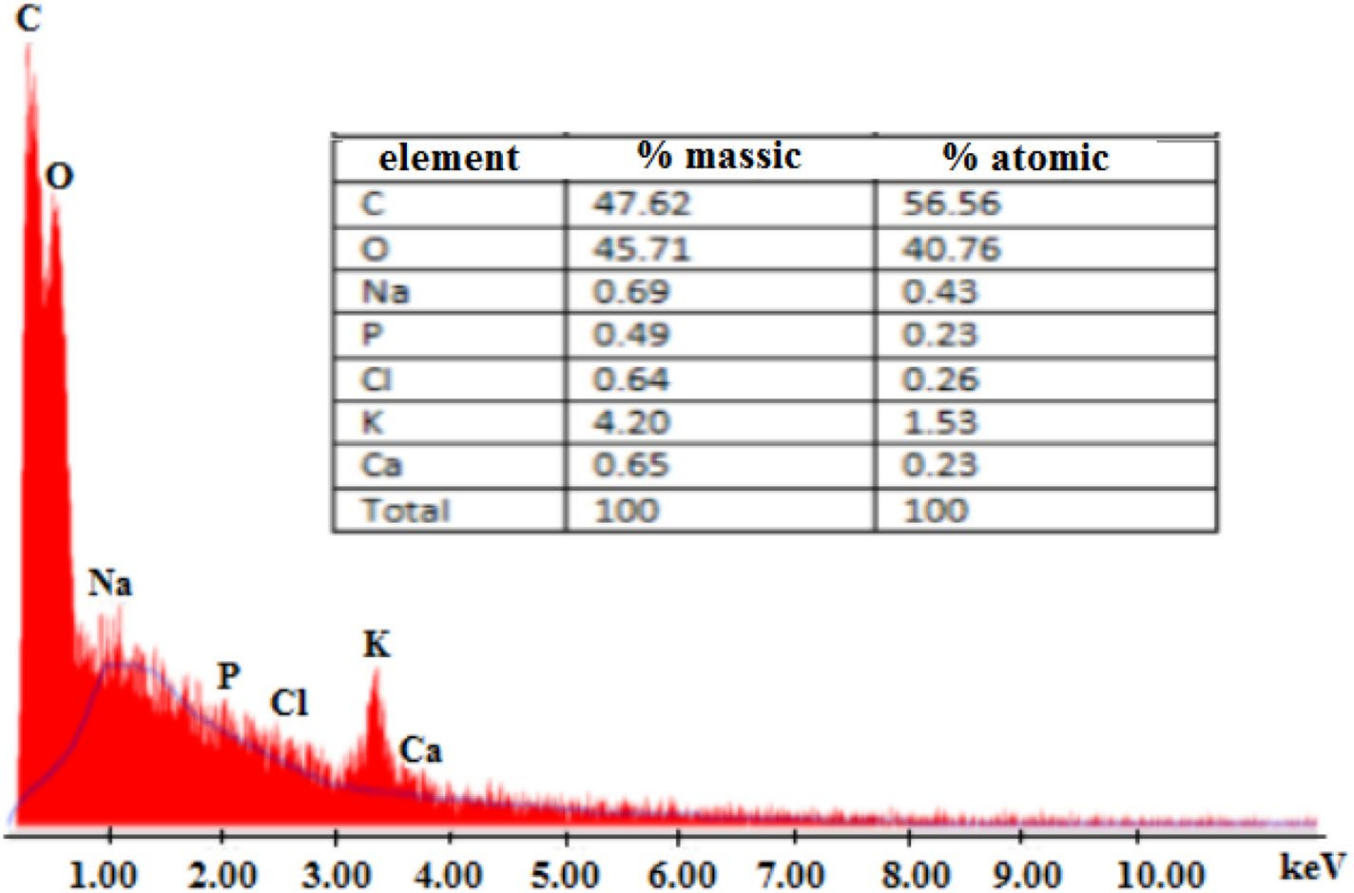
EDX spectrum and elemental analysis of potato powder (PP).

The elemental compositions were analyzed by EDX (energy dispersive X-ray microanalysis). Although the structure of biomaterials is mainly made from cellulose, hemicelluloses and lignin, which are generally similar in structure, the type of functional groups and their acid and basic properties are different. The Energy Dispersion Spectroscopy X (EDX) of potato powder (PP) presented in Fig. 2 shows the presence of different elements such as carbon, oxygen, sodium, potassium, etc. The two major elements detected in PP before adsorption process were carbon by weight (47.62%) and by atom (56.56%), and oxygen by weight (45.71%) and by atom (40.76%), as shown in the table of Fig. 2. It is clear that the proportions of carbon and oxygen are the highest, which confirms the organic nature of the adsorbent material.

### Morphologic analysis

**Figure 3:**
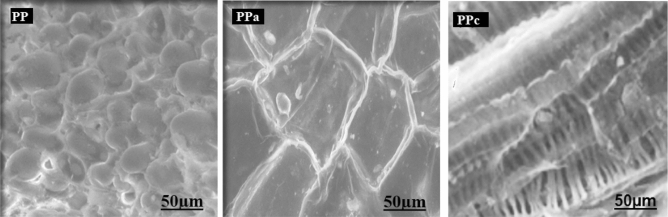
Scanning electron microscopy SEM micrographs of the native, activated and calcined material before treatment.

Several works were done to characterize the biolignocellulosic material by SEM for a better understanding of the physical–chemical and morphological characteristics^30, 31^. The observation by a scanning electron microscopy (SEM) (Fig. 3) shows micrograph of native and activated sorbents, where the pores in PPa are larger than in PP case.

While micrograph of active sorbent (PPa) indicates that the external surface of the activated material displays a series of cavities with different dimensions for the material distributed over the surface. The micrograph of calcined sorbent (PPc), illustrates the surface morphology of the substrate after calcination. It clearly shows the presence of PPc nanotubes, which appear to be well ordered. This nanoporous structure can facilitate the anchoring and nucleation of the dyes on the PPc substrate.

### Thermogravimetric analysis

**Figure 4:**
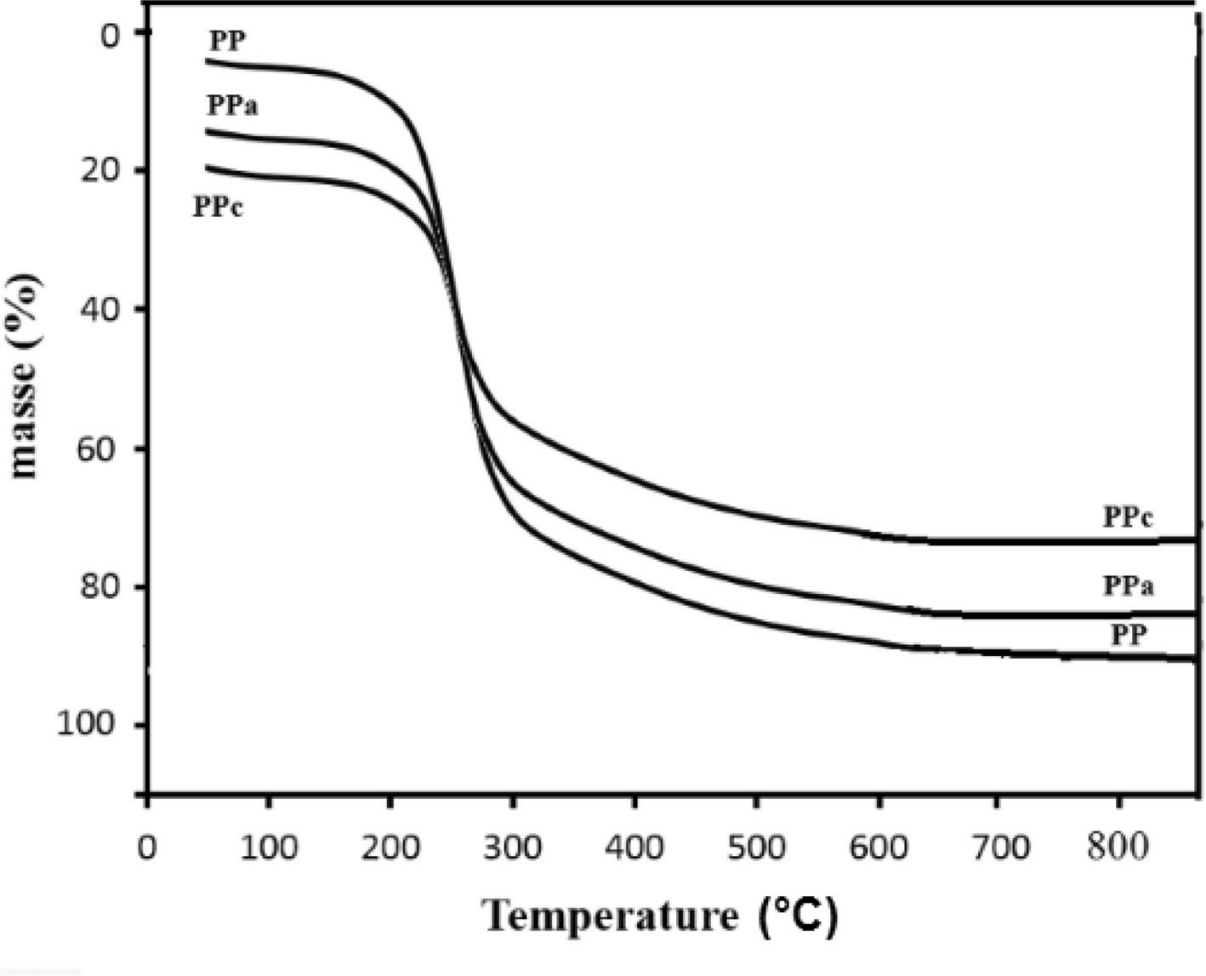
Thermogravimetric (TG) curve of the native, activated material and calcined material.

For thermogravimetric analysis (TGA), 20 mg of samples were introduced in a platinum sample holder and heated at a rate of 10° / min under air flow of 100 mL/min up to 800 °C on a TGA92 instrument from Setaram (Lyon, France)^1, 2^. The TG curve of the potato peels powder (PP) ( Fig. 4) shows a continuous weight loss at about 220 °C related to moisture release (the loss of water), which is adsorbed both on the surface and in the pores of the sorbents. The second mass loss step in the temperature range 200–400 °C represents the decomposition of cellulose and Hemicellulose^21, 22^.

At temperatures higher than 400 °C, the loss of biomass may be indicative of lignin weight loss^23^. The TGA graphs were superimposed using the dry weight at 220 °C as origin. This reveals that the mass loss due to lignin combustion increases as the mass of lost water decreases as illustrated in Table 2.

**Table 2:** Inverse relation between water contents and adsorbed lignin contents.

Adsorbants	Mass loss lignin %	Mass loss water %
PP	90	5
PPa	83	17
PPc	73	20

### FTIR analysis

**Figure 5:**
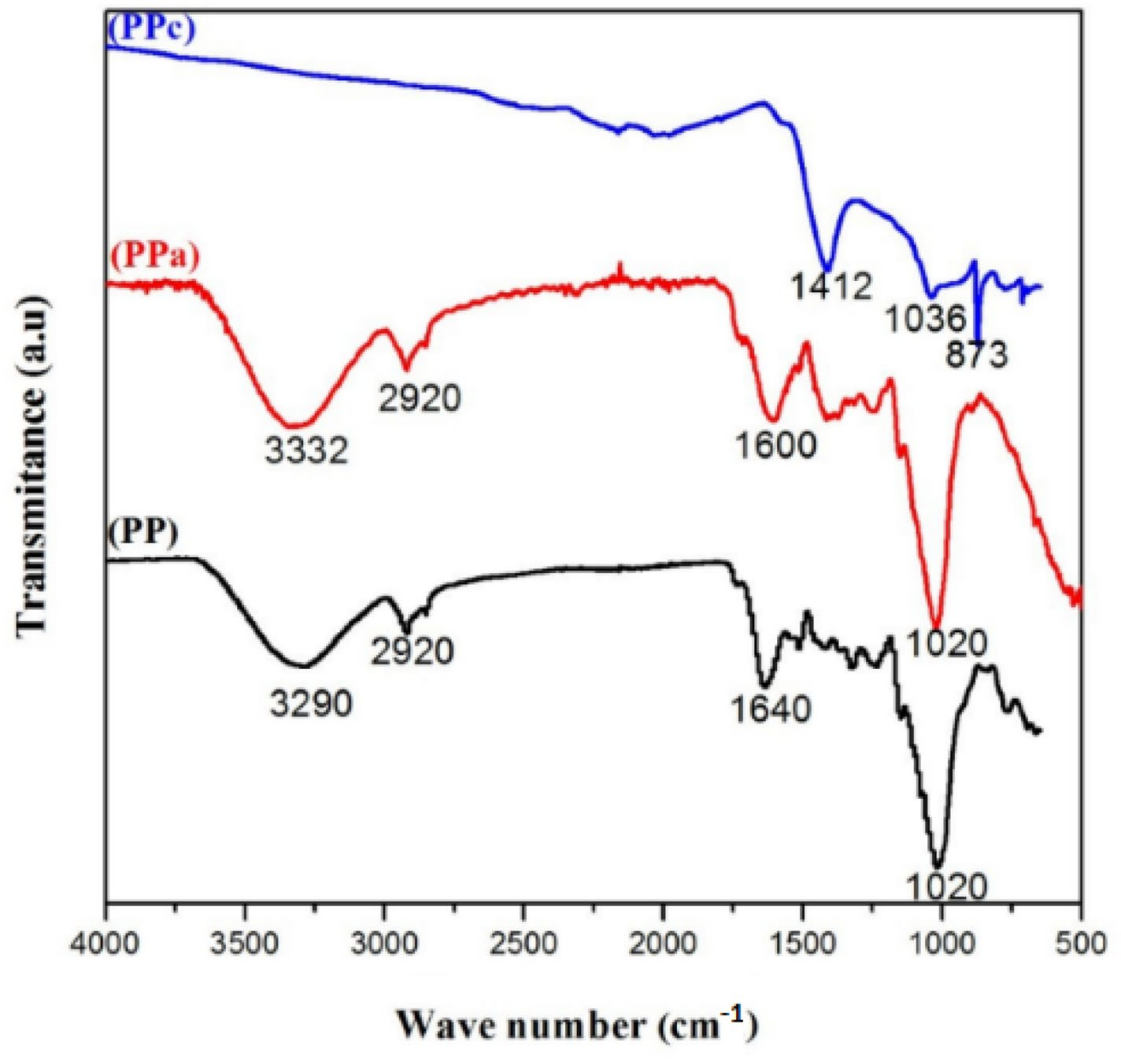
FTIR spectra analysis of native, activated and calcined materials.

Infrared spectra were recorded using the KBr technique with a Mattson Genesis II spectrometer^1–7^. The spectra of potato peels powder were measured by an FT-IR spectrometer within the range 500–4000 cm^−1^ to determine the vibration frequency in the functional groups. The spectra of the native PP and activated material PPa are quite similar (Fig. 5). In the high frequency region of the spectrum, bands at 3000–3600 cm^−1^, the tailing towards lower frequencies is due to OH stretching modes of H- bonded hydroxyl groups, while several weak bands between 2960 and 2850 cm^−1^ can be assigned to saturated CH stretching modes. The bands at 1600 cm^−1^ are the combination of C=C stretching vibration of the aromatic ring structures and conjugated systems such as diketone, ketoester, quinone (1550–1680 cm^−1^). The adsorption bands between 1000 and 1050 cm^−1^ may result from vibrations that show bands at 1010 and 1020 cm^−1^ (C-O stretching).

The primary differences in the FTIR spectra of the activated carbons, after the pyrolysis at high temperature (800 °C), are shown in the regions 1400 and 1040 cm^−1^. For PPc, the bands at 1412 cm^−1^ is due to the skeletal (C=C) vibrations of aromatic rings and the band 1036 cm^−1^ is assigned to C–O–C lactone structures (stretching C–O vibrations).

### Effect of pH

**Figure 6:**
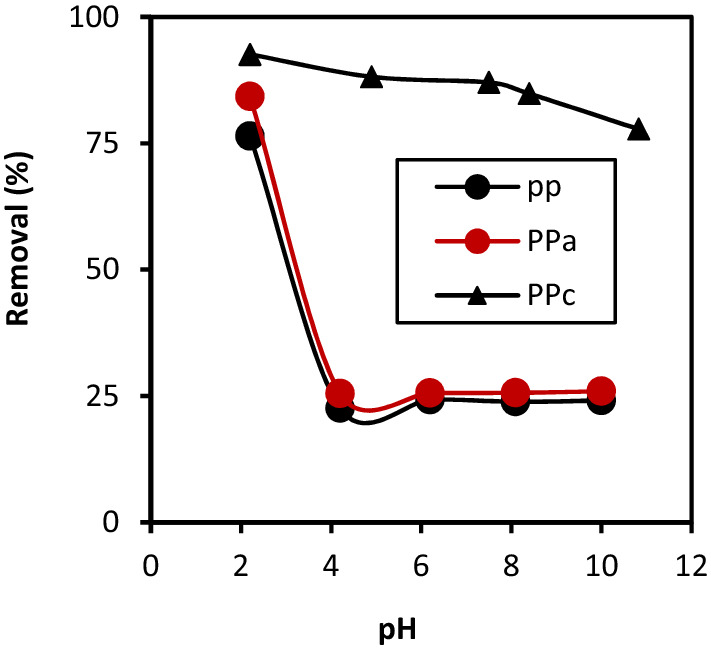
Effect of initial pH on CB adsorption of native, activated and calcined materials (C_0_ = 30 mg/L; m (PP) = m (PPa) = 2 g/L; m (PPc) = 0.6 g/L; T = 25 ± 2 °C).

The pH plays an important role in the adsorption of any dye onto materials. Therefore, the first factor investigated in the present work is the effect of pH on CB removal. Figure 6 shows the aforementioned pH-effect. The CB removal at strong acid pH values was found to be very large. The optimum pH for CB sorption by all sorbents was ~ 2.2: (76.41, 84.26 and 92.6% for PP, PPa and PPc, respectively). At lower pH, the surface of the adsorbent becomes more positive promoting electrostatic attraction activities between the negatively charged SO_3_¯ anion of the dye and the adsorbents. Except for PPc, as the pH increases from 2 to 10, there was a steady decrease in the amount of CB being adsorbed, revealing thereby that PPc was acidic in the pH range 2–10. There was little effect on the removal of CB in the range of 2.2 to ambient pH (92.6% at pH 2.2 to 86.3% at pH 7.5). This suggests that CB molecules are not adsorbed through the ionic SO_3_¯groups^24^. Hence, the ambient pH was chosen for the study of CB sorption isotherm on PPc. This result does not agree with that reported by Kyzas^25^, where the carbonization of potato peels at 800 °C caused a collapse in the texture of the activated carbon prepared. The total elimination for PPc is observed for a capacity of 46.3 mg g^−1^ at fixed equilibrium time (2 h). Furthermore, unlike the native material (PP), elimination is partial; we obtain an adsorption capacity of 11.46 mg g^−1^.

### Kinetic studies

**Figure 7:**
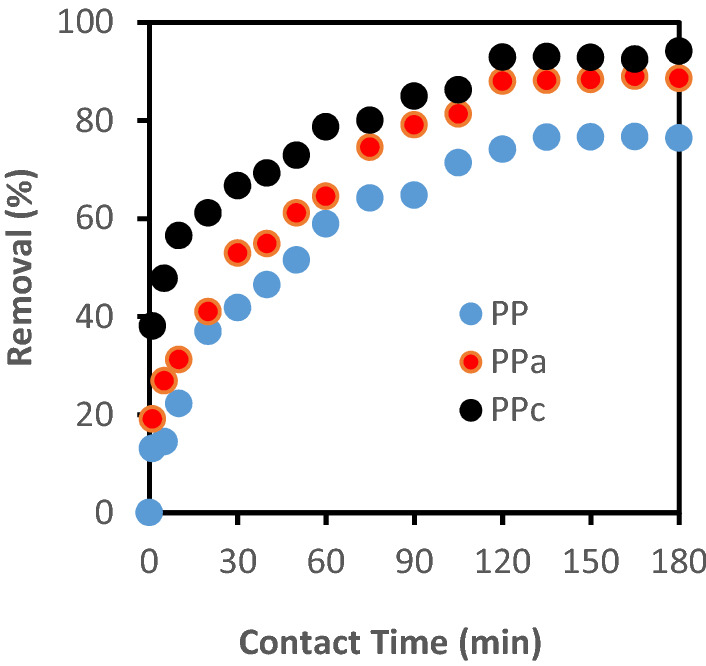
Effect of contact time on the sorption of CB onto native, activated and calcined sorbents at pH = 2.2 (C_0_ = 30 mg/L; m (PP) = m (PPa) = 2 g/L; m (PPc) = 0.6 g/L; T = 25 ± 2 °C).

Experimental kinetic data for adsorption of CB onto native PP and activated (PPa, PPc) adsorbents for a 30 mg L^−1^ dye solution, at pH 2.2 are illustrated in Fig. 7. The total elimination at equilibrium adsorption time (180 mn), is 94%, 88.6% and 76.41% for PPc, PPa and PP respectively. Two simplified kinetic models including pseudo- first-order and pseudo-second-order equations were used in this study.

The results of the kinetic parameters based on the values of the correlation coefficients R^2^ are shown in Table 3. The data fit better with the second order kinetic model than with the pseudo first order for the three adsorbents. Thus, the adsorption rate is influenced by the availability of the adsorption sites rather than by the concentration of the dye in the solution^26^.

**Table 3:** Parameters of kinetic equations for the adsorption of CB onto PP, PPa and PPc.

Adsorbent	qe, exp (mg g^−1^)	Pseudo-first-order model			Pseudo-second-order model		
		qe, cal (mg g^−1^)	k_1_ (min^−1^)	R^2^	qe, cal (mg g^−1^)	k_2_ (g mg^−1^ mn^−1^)	R^2^
PP	11.50	5.2456	0.0226	0.318	13.33	0.0027	0.985
PPa	13.30	17.336	0.0820	0.753	15.015	0.0029	0.984
PPc	47.00	27.05	0.0437	0.960	49.26	0.0018	0.993

The q_e_ values for PP, PPa and PPc obtained from the pseudo-second-order rate model were in better agreement with qe (exp) values compared to those obtained from the pseudo-first-order rate model. The pseudo second order kinetic model is based on the assumption that the rate-controlling step may be a chemisorption involving valence forces through sharing or exchange of electrons between biosorbent and sorbate^27^.

### Sorption isotherms modeling

**Figure 8:**
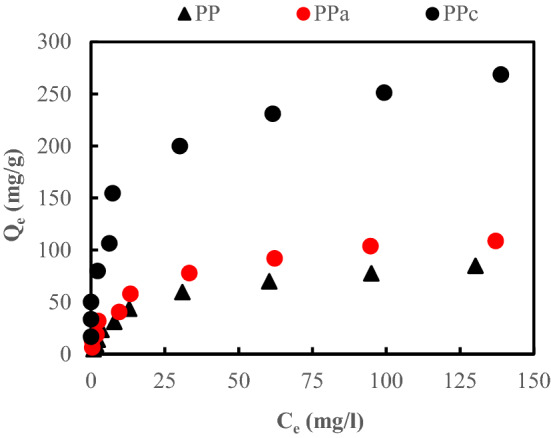
Sorption isotherms of CB for the native and modified sorbents (m (PP) = m (PPa) = 2 g/L; m (PPc) = 0.6 g/L; pH = 2.2).

The adsorption isotherms of synthetic aqueous solutions of CB on PP, PPa and PPc at variable initial concentrations of dye is displayed (Fig. 8). According to the classification of Giles and Col^28^, the adsorption isotherms of the CB dye are of type L. The comparison of the adsorption capacities is of the following order (PPc > Ppa > PP). The isotherms follow Langmuir's model perfectly. Therefore, the attraction forces between the adsorbed molecules are low^28^. The experimental data of adsorption equilibrium were tested by means of the Langmuir (type I, type II) and Freundlich isotherms. Based on the corresponding correlation coefficient R^2^ values and the adsorption capacity estimated by the models shown in Table 4, it was found that the Langmuir II model gives the best fit for all the sorbents. The value of 1/n is within the limit range of validity of (0.3–0.5), and this confirms that the isotherm is of type L.

**Table 4:** Parameters for different adsorption isotherm models (m (PP) = m (PPa) = 2 g/L, m (PPc) = 0.6 g /L; pH = 2.2).

Isotherm model	Parameters			
Langmuir I	q_exp_	q_th_	K_L_ (L mg^−1^)	R^2^
PP	85.0	125.0	0.048	0.990
PPa	108.6	58.8	0.460	0.976
PPc	268.5	238.1	0.211	0.921

Isotherm model	Parameters			
Langmuir II	q_exp_	q_th_	K_L_ (L mg^−1^)	R^2^
PP	85.0	100.0	0.063	0.996
PPa	108.6	125.0	0.088	0.990
PPc	268.5	270.3	0.215	0.992

Isotherm model	Parameters			
Freundlich	K_f_ (mg g^−1^)	1/n	R^2^	
PP	2.504	0.531	0.924	
PPa	3.257	0.427	0.932	
PPc	70.360	0.284	0.936	

Considering the effect of calcinations (carbonization), PPc exhibits the best adsorption capacity and the higher value of K_L_, compared to the other biomaterial according to Langmuir II. The maximum sorption capacity q_max_ of CB dye is 270.3 mg g^−1^; the high value of Langmuir constant K_L_ (0.215 L mg^−1^) suggests a strong bonding of the CB dye due to the adsorption free energy and the specific adsorbent affinity. Its value is the reciprocal of the CB dye concentration at which half of the saturation of the adsorbent is attained.

## Conclusion

The adsorption experiments indicated that Waste Potato Peels Powder (PPc) calcined at 800 °C was effective in removing greater than 94% of CB dye from aqueous solution within 180 min at pH = 2.2. The kinetics of adsorption conformed to a pseudo-second-order model indicating a chemisorption adsorption process. Isotherm studies were well described by the Langmuir, confirming the single-layer, homogeneous and chimisorption adsorption process. The experimental results have shown that PPc (270.3 mg g^−1^) has a greater capacity compared to pp (100 mg g^−1^) and ppa (125 mg g^−1^). Physical activation of sorbent Waste Potato Peels Powder results in enhanced sorption capacity for CB.

Therefore, Waste Potato Peels Powder is a low cost, eco-friendly and a potential alternative adsorbent to high cost commercial adsorbents for the treatment of dye-contaminated wastewater.

The drawback of the given processes is the high sludge production formed for Wastewater treatment. Sludge treatment has become one of the most important environmental issues. Thus, the use of biochar as an addition to sewage sludge can be a new and interesting solution in future utilization of sewage sludge.
